# Median Neurectomy1Read before the March meeting of the New York County Veterinary Medical Association.

**Published:** 1899-05

**Authors:** C. E. Clayton

**Affiliations:** Assistant Surgeon, American Veterinary College, New York


### MEDIAN NEURECTOMY.[1]

By C. E. Clayton, D.V.S.,
ASSISTANT SURGEON, AMERICAN VETERINARY COLLEGE, NEW YORK.

Before entering upon the operation, its results, the indications and contraindications, let us briefly review its history. C. Pellerin, in his little work on this subject, which was translated by Prof. Liautard for our benefit, states that to the best of his knowledge

[1] Read before the March meeting of the New York County Veterinary Medical Association.

Peter, of the Berlin Veterinary School, was the first to perform it, and read an article on the subject December 2, 1885, before the Society of Veterinary Practitioners of Berlin. Then followed Ries, of Ettelbruck, in an article entitled "A Veterinary Excursion in Belgium," in which he asks the following questions: Is gangrene, as an accident subsequent to neurectomy, the termination of a more or less severe laminitis, or is chronic laminitis a special complication of section of the median nerve? Again in 1893, Kull, a German, reports good results on a horse that suffered with ringbone and a disease of the foot not named. In 1894 Baldoni, of the Milan School, published a pamphlet upon this operation, and again in 1894 Pellerin issued an article on new cases which he had operated upon since his article of 1892.

So much for the history, which is quite brief, but sufficient for our purpose, and for which we are greatly indebted to C. Pellerin and Prof. Liautard for the translation. Let us now take a brief review of the surgical anatomy of the parts upon which the operation is performed, which includes the skin of the axilla, lower part of the sterno-aponeuroticus and its aponeurosis, antibrachial fascia, superior extremity of the radius, internal flexor of the metacarpus, artery, vein, and nerve—the last three being the most important. The nerve, as you all know, has its origin from the eighth cervical and first and second dorsal pairs, and leaves the posterior part of the brachial plexus, passing under the axillary artery, to gain the front of the humeral artery, which it follows to its termination, which is from before backward and from above downward. It then accompanies the posterior radial to the humero-radial articulation, and then crosses over outwardly the latter artery to become posterior to it, in which position it continues down the leg, but not so deeply situated as the artery. At its arrival at the above articulation it gives branches to the internal flexor of the metacarpus and to the two flexors of the phalanges. The artery has been sufficiently described when describing the nerve, and it will also do for the vein which is formed from the metacarpal veins and always accompanies the artery and nerve; these relations you will find to be the most constant.

The instruments required are two scalpels or bistouries, curved scissors, blunt retractors, plain dissecting forceps, director, aneurism hook, syringe, and cocaine. The animal is cast upon the side upon which the operation is performed. Any hobbles will do that allow of removing one of the legs, upon which the side-bar is placed, the other end to the coronet or shin of one of the legs which is still fast in the hobbles. I should here state that before casting I inject cocaine at the seat of operation, and by the time you are ready to operate, anæsthesia of the skin and subjacent tissue is complete; now clip hair as close as possible and sterilize the skin, the instruments having been previously sterilized.

Let us now proceed to the operation, and I wish to state that the neatness and rapidity of performing the operation depend wholly on where the incision is made. This remark will probably bring to our mind where we are to make the incision and by what landmarks, so to speak, are we enabled to decide upon the exact location. Without reference to others, I will try to make plain how to select the place. Place the finger at a point between the postero-internal border of the radius, which can be plainly felt, and chestnut, which is on the inside of the forearm and behind the radius, pass the finger upward in a vertical direction, which you will see does not run parallel to either the front or back of the forearm; when arrived at the axillary space, where the skin is very much wrinkled, you will feel the finger pass over a ridge, which will be the inferior border of the sterno-aponeuroticus. Commencing at about one inch above this inferior border of the muscle, make an incision of about one and one-half inches in a downward direction, and on the imaginary line which you have traced; the incision will be sufficiently large if made according to these directions. This will expose the muscle already named, which is divided in the direction of its fibres, which are downward, revealing the loose cellular tissue which is here abundant; this is torn loose by forceps or the handle of the scalpel, but preferably cut, as there is less thickening left when the wound is healed than when done by the tearing process. Where it is possible in any operation I prefer to cut in preference to tearing, as wounds heal much more quickly and with less cicatrix. We now have exposed the antibrachial fascia, which is attached to the posterior border of the radius; make a slight puncture in this about one-quarter of an inch behind the radius, pass the director in this opening, and with scalpel or blunt-pointed bistoury enlarge the opening the same size as the one in the skin. Let me caution you to follow this mode of opening the aponeurosis, and thereby avoid a rather annoying complication which I once experienced, which was a violent struggle on the part of the animal, and the scalpel went in too deep and punctured the vein, and the flow of blood very much interefered with the operation by obscuring the field. With your blunt retractors draw back the edges of the wound together with the internal portion of the internal flexor metacarpi with plain dissecting forceps, and by no means use rat-toothed or bull-dog forceps, as they are called, as you might puncture the vein, which is annoying.

As before stated, isolate the nerve, but first find it and know it when you see it. This ought to be easy if it was always found where the books say it is. My experience has led me to believe that if there is any one place in the body where relations differ it is here. Sometimes it is on top, in plain view as soon as the lips of the wound are retracted; again in front of the vein; again behind it, and still again beneath it, and on one or two occasions it was beneath the artery, though this latter situation is very rare; but one thing is absolutely certain, and that is, if you have made your incision as directed and you do not see the nerve at first, it is only a matter of looking deeper down in the space between the posterior face of the radius and anterior surface of the internal flexor metacarpus, as it is always here unless some one else has removed it. Now having isolated it, pass the aneurism hook underneath, allowing the free ends of the hook to rest on the skin at each side of the wound. This will hold the nerve up and allow the severing of it by scissors or any of the neurotomy knives; then pick up the distal end of the nerve and cut off about an inch. This completes the operation for me, as I do not stitch the lips of the wound or attempt to apply a dressing to the part. The animal is now allowed to rise, and trotted, and if a fit subject for the operation the result is nothing less than marvellous.

Having described the operation, perhaps, too much in detail, although I think not, for by so doing I have tried to warn you so that you may not experience the annoying parts which I have been through, we will now consider the diseases giving rise to lameness which can be permanently or partially relieved. As the median nerve supplies the inside of the forearm and leg, and as we have excised a part of it at the superior extremity of the radius, sensation must have been destroyed or greatly lessened in all that part below the section, and here we find knee, shin, fetlock, pastern, coronet, foot, and tendons.

I have operated upon a number with great success where there were speedy cuts which had caused a bony growth; but where anchylosis of the entire carpus exists it will avail nothing; splints of all sizes, situations, and extent with more than gratifying results. One case in particular, where the splint extended from the inside to the outside, had been fired three times and blistered as many more, and still lame if worked, was operated on July 5, 1897, and put to work on August 5, 1897, going sound, won a prize in every class he was entered in at Madison Square Garden that fall, which was three, sold for fifteen hundred dollars, worked all last season at Newport, and turned out last fall, and has not taken a lame step on the leg operated upon.

Bony growths at the inside of the fetlock are afforded positive relief, unless, as in the knee, there is anchylosis; the same with the coronet. In cases where we have side-bones it is very difficult to determine which one is causing the lameness, as I have found that it need not of necessity be both, but only one which gives rise to the lameness. This can be detected by injecting cocaine over the inside plantar nerve, and if the lameness disappears median neurectomy is of great service.

In navicular disease it is far more preferable in heavy draught-horses than plantar neurectomy, as it will relieve the lameness sufficiently to enable the animal to work, and has the advantage of not removing all sensation from the foot. Of course, it would not be as effectual in driving horses, but then, perhaps, when not too bad, it might be resorted to in them, and if not lasting in the relief afforded it would be only a simple matter to excise the outside plantar nerve and make digital neurectomy complete. The first case I operated upon was on December 24, 1896, and I have yet to record any deleterious results which could be traced to the operation, so that Ries's questions, which are given in the early part of this paper, have been satisfactorily disposed of.

Baldoni states that he has seen cases of shoulder-lameness cured by this operation, but I can only agree with C. Pellerin when he says that those cases must have been ones of mistaken diagnosis.

The value of this operation for the relief of lameness, which has resisted firing and blistering several times, appeals to me most strongly from my experience with it, as I am positive that many an animal has been destroyed as incurable that might have been of service for many years had median neurectomy been performed.

